# Validation of the Cognitive Assessment of Later Life Status (CALLS) instrument: a computerized telephonic measure

**DOI:** 10.1186/1471-2377-7-10

**Published:** 2007-05-21

**Authors:** Valerie C Crooks, Thomas D Parsons, J Galen Buckwalter

**Affiliations:** 1 Department of Research and Evaluation, Kaiser Permanente Southern California, 100 S. Los Robles, Pasadena, CA 91101, USA; 2 Institute for Creative Technologies, University of Southern California, 13274 Fiji Way, Office 301, Marina del Rey, CA 90292-4019, USA; 3 eHarmony, 300 N. Lake Ave., Suite 1111, Pasadena, CA 91101, USA

## Abstract

**Background:**

Brief screening tests have been developed to measure cognitive performance and dementia, yet they measure limited cognitive domains and often lack construct validity. Neuropsychological assessments, while comprehensive, are too costly and time-consuming for epidemiological studies. This study's aim was to develop a psychometrically valid telephone administered test of cognitive function in aging.

**Methods:**

Using a sequential hierarchical strategy, each stage of test development did not proceed until specified criteria were met. The 30 minute Cognitive Assessment of Later Life Status (CALLS) measure and a 2.5 hour in-person neuropsychological assessment were conducted with a randomly selected sample of 211 participants 65 years and older that included equivalent distributions of men and women from ethnically diverse populations.

**Results:**

Overall Cronbach's coefficient alpha for the CALLS test was 0.81. A principal component analysis of the CALLS tests yielded five components. The CALLS total score was significantly correlated with four neuropsychological assessment components. Older age and having a high school education or less was significantly correlated with lower CALLS total scores. Females scored better overall than males. There were no score differences based on race.

**Conclusion:**

The CALLS test is a valid measure that provides a unique opportunity to reliably and efficiently study cognitive function in large populations.

## Background

The human and economic costs of cognitive decline and dementia [1] point to the need for an improved understanding of age-related cognitive deficits and the prevalence of such deficits in the United States [2,3]. The distinction between normal age-related changes in cognition from deficits indicative of incipient dementia is problematic but increasingly relevant in health care research and epidemiological studies.

Current standards of good practice entail that the assessment of cognitive performance to differentiate between age-related neurocognitive deficits and neurodegenerative disorders be conducted by neurologists and by clinical neuropsychologists who use standardized neuropsychological testing [4-6]. Exhaustive neuropsychological batteries, however, tend to be limited to specialized medical centers. While some briefer evaluations (such as the CAMCOG, CAMCOG-R) have been developed that contain multiple cognitive domains, they require in-person administration [7,8]. As a result, primary care physicians are the most likely observer of cognitive change among older persons. Studies have shown, however, that physicians often underreport or fail to identify problems with cognition [9,10]. Strategies that improve the availability of cognitive measures that may assess multiple cognitive domains are timely and appropriate.

Although a variety of cognitive screens (in-person and telephonic) have been developed to monitor cognitive decline [11-17], they tend to contain only a few limited measures of cognitive functioning [12]. The Mini-Mental State Exam (MMSE) [18] is the most widely used in-person instrument for assessing cognition among older adults. The MMSE has shown good test-retest reliability (0.89) and inter-rater reliability (0.82) [18]. The MMSE, however, has failed to demonstrate consistent predictive ability among heterogeneous populations. It shows a high rate of false positives among individuals with low socioeconomic status and low education [19-21] and false negatives in mildly impaired persons [22-24].

Telephone screening tests of cognition have been developed. The most frequently used is the Telephone Interview of Cognitive Status (TICS) [25] and, an adapted version that adds a delayed memory item, the TICS-modified (TICSm) [26]. The TICS was originally adapted from the MMSE. The TICS and the TICSm show high correlations with the MMSE [25,27] and equivalent sensitivity and specificity as cognitive screens [28]. While the TICS and TICSm share the MMSE's strength as a general screening measure, these screening tests also share the MMSE's potential for failing to detect subtle cognitive decline, and cannot substitute for neuropsychological assessment in answering questions of cognitive decline [22]. The fact that these screens do not measure many of the cognitive domains used in a full neuropsychological battery is a major limitation. As a result, great caution needs to be exercised in using such screens because they are often unable to proffer reliable information about specific cognitive domains affected [29]. Hence, there is need for a reliable and more comprehensive tool.

To this end, a psychometrically valid, time-efficient, telephone-administered test of cognitive performance associated with aging, the Cognitive Assessment of Later Life Status (CALLS), was modeled after standardized neuropsychological batteries to overcome the limitations of screening batteries modeled on the MMSE and in person administration. The objective of this study was to validate the CALLS instrument.

## Methods

The study was reviewed and approved by the Institutional Review Board of Kaiser Permanente Southern California.

### Instrument development

The CALLS instrument has undergone extensive developmental work. Applying classical psychometric theory, we have followed a sequential, hierarchical strategy for developing this test, where each stage of development does not proceed further until specified criteria are met. A brief explanation of preliminary work to the validation study follows.

### Item generation stage

A range of cognitive items were identified as necessary to be included for an effective telephone screen. These comprehensive cognitive items were pilot tested with 43 elderly participants (mean age = 73.2; female = 56 percent; non-white = 41 percent) over six separate telephone testing sessions. These cognitive domains were correlated with a brief battery of in-person neuropsychological tests (Judgment of Line Orientation, Boston Naming Test, Letter Number Sequencing, Trail-Making Test Parts A and B and the California Verbal Learning Test). Four separate focus groups were held with participants to elicit feedback on the comprehension and clarity of the questions and test experience. An Expert Panel, comprised of a team from neuropsychology, psychometry, geriatrics, speech pathology, audiology and epidemiology were consulted and a core set of items with acceptable face validity, usability and preliminary convergent validity were identified. Tests of verbal learning and memory, attention and working memory, orientation, processing speed, and executive functioning, along with assessment of auditory discrimination and depression were considered essential.

### Item selection stage

Based on the selected cognitive items, the prototype CALLS test was administered to 101 participants over the age of 64, randomly selected from the membership of Kaiser Permanente Southern California. All participants took part in two 30-minute test sessions over the telephone. The reliability of the subtests was good with coefficient alphas between 0.72 and 0.87. Principal component analysis was conducted to evaluate construct validity. Resulting components were derived from tests of verbal learning and memory, attention and working memory, executive functioning, and verbal fluency and naming. Coefficient alpha for the final set of items was 0.77. The Expert Panel agreed that those with high factor loadings be retained and those without be eliminated. A 30 minute interview was created through streamlining of instructions, use of adaptive questions and skip patterns.

### Validation stage

To validate the test, each participant was given the 30-minute telephone CALLS battery and a full 2.5 hour in-person neuropsychological battery of tests. Approximately half of the final sample was given the CALLS interview first (n = 108) and half were given the neuropsychological interview first (n = 103). Every effort was made to ensure that the two tests were administered within a reasonably close time period without fatiguing the participant. The mean time between tests was 16.27 days (Range: from one day to 60 days).

### Current study

#### Sample

No participants from previous interview pools were recruited to subsequent interview pools. In the validation study, 908 men and women 65 years and older were randomly selected from the membership of Kaiser Permanente Southern California. Sampling was conducted to maximize the chances of ethnic and racial diversity. Based on geocoding, equal numbers of African -Americans, Whites, Hispanics and Asians were sought. Also equal numbers of men and women were targeted. Once the target criteria for a given ethnic or racial group were met, no more participants were recruited for that group. Each participant was recruited by initially sending a letter which described the nature of the study and provided an opt-out postcard and study brochure. Due to the time commitment and diversity goals, participants were provided with an $80.00 incentive for participation in the CALLS interview and the in-person neuropsychological assessment. A maximum of six calls were made to recruit for the study.

Of the original 908 person sample, 152 were excluded due to ineligibility (125 language barrier; 10 deaths; 6 under 65; 5 illness; 3 each for severe hearing problem and relocation out of area). From an eligible pool of 756, a total of 211 consented to participate (response rate 28 percent) to both the in-person neuropsychological battery of tests and the 30-minute CALLS battery. Each participant signed an Institutional Review Board approved consent form prior to taking the in-person neuropsychological test battery. The breakdown of the final sample selection is described in Table 1.

There were no significant mean age differences between participants (mean = 73.4 years; SD = 5.8) and non-participants (mean = 72.8 years; SD = 6.4). As shown in Table 2, the sample was evenly divided between men (49 percent) and women (51 percent) in both groups. Hispanics were about twice as likely to be non-participants (36 percent) than participants (19 percent). Asians were slightly more likely to be non-participants (28 percent) than participants (21 percent). Contrariwise, Whites were about two and a half times more likely to be participants (36 percent) than non-participants (14 percent). There were no differences in groups for African-Americans (p < 0.0001).

As shown in Table 2, the participant group is well represented in terms of age, gender and racial and ethnic groups. The study sample is slightly better educated than the general population in these age ranges, but over one quarter have a high school education or less.

### Cognitive measures

Lay interviewers (with at least a bachelor's degree) were trained and supervised by a neuropsychologist to conduct the standardized in-person neuropsychological test battery. Lay interviewers were also trained by a neuropsychologist and supervised by project staff in conducting the standardized CALLS telephone test. Analysis of the reliability of the interviewers' performance across testing sessions revealed correlations in an acceptable range from 0.75 to 0.86.

### CALLS telephone test

The CALLS test includes many of the same cognitive items as are used in a neuropsychological battery. It also includes items that measure response time. The CALLS is a computer-assisted test that is standardized with precise scripts and cues for interviewers. The program is designed to not proceed to the next question item until a valid response is entered. Animal Naming, F Words, and Similarities are audio recorded for post-test scoring to ensure that all responses are entered correctly and in the order given.

Test items that are similar to existing tests include: *Date *– Month, day, date, season and year; *President/Vice President *– name current; *Serial Backward 7 *– Subtract 7 from 100 up to 5 times; *Digit Span Forward *– Digits given from 3 to 7 digits; *Digit Span Backward *– Digits given from 2 to 6 digits; *Animal Naming *– 30 seconds to name animals; *F Words *– 30 seconds to name F words.

Other test items are similar to existing tests but include new word lists, naming and similarities. These include: *Naming *– 4 questions with brief descriptions are asked to identify number or objects (answers: dozen, umbrella, bed and elephant)*; Three Trial Wordlist *– 12 words across 3 trials with immediate recall (wordlist: brother, steel, day, cousin, month, copper, second, niece, brass, mother, silver, minute);*Wordlist Recall *-1) recall all 12 words remembered after delay with other tests; 2) recall when cued (prompted with "Tell me any words from list related to: metals, relatives, and units of time"); 3) recall wordlist with intrusion words (12 incorrect words are added to correct list, prompted with "Tell me if the word was on the original list of 12 words); and *Similarities *– 4 pairs of similarities (prompted with "How are a hammer/saw, skirt and pants, fruits and vegetables, bus/car alike?").

New and unique features and tests include: *Volume Configuration *– A range of 4 different volume choices are tested and selected by the participant prior to the main CALLS interview; *Pitch Discrimination *– Participant is given 15 paired tones in a row and must distinguish whether the two tones or pitch are the same or different; *Simple Reaction Time *– A series of tones are played at random intervals for right and left ears. The participant will be presented with a target tone. Each time the tone is heard, the participant must verbalize "now" as quickly as possible when the target tone is heard; *Choice Reaction Time *– Participants listen to a target tone and identify that tone from a series of tones with varying pitch. Participants distinguish tones by responding "now" for correct tone within 20 specific tone sequences (5 each high and low, 10 medium). All tones are computer-generated and practice tests are performed prior to actual tests. Response time items are recorded and time stamped to the millisecond to ensure accuracy.

Non-cognitive tests included a 20-question adaptation of the Center for the Epidemiological Study of Depression (CESD) [30] and a brief hearing survey regarding phone use, use of amplifier, and hearing aids. Interviewers completed a feedback questionnaire to evaluate protocol adherence, hearing assessment, and attitude of interviewee.

### In-person neuropsychological battery

The in-person tests chosen to compare with the CALLS are well standardized and have acceptable reliability and validity. Verbal memory was assessed with the paragraph prose recall tests from the Wechsler Memory Scale-III: Logical Memory I, Logical Memory II, Logical Memory Recognition (WMS III) [31] and the California Verbal Learning Test (CVLT) [32]. Nonverbal memory was assessed with the Faces I and II test from the WMS III [31]. Attention was assessed with the Digit Span Forward test from the WMS-III [30] and with the Trail-Making Test Part A [33]. Working memory was assessed with Letter-Number Sequencing and Digit Span Backward from the WMS-III [31]. Visuospatial perception was tested with the Judgment of Line Orientation (JLO) [34]. The Trail-Making Test Part B [31] and the Controlled Oral Word Association Test (Phonemic Fluency – F-A-S) [35] evaluated executive functioning. Verbal fluency and naming were tested with the Boston Naming Test (BNT) [36] and Animal Naming (Semantic Fluency) [37]. One hundred and ninety-seven participants were given the Mini-Mental Status Exam [18].

Additional tests conducted in-person but not analyzed here included the Wechsler Test of Adult Reading (WTAR) [38], Symptom Checklist-90 (SCL-90) [39], Geriatric Depression Scale (GDS) [40], and the Lubben Social Network Scale – Revised (LSNS-R) [41]. Interviewers were trained by an audiologist and administered standard audiology tests to assess hearing during the in-person interviews.

### Statistical analyses

Descriptive statistics (t-test, chi squares) were generated for demographic characteristics of the sample (Table 2) and mean scores for CALLS (Table 3) and neuropsychological assessment data were calculated.

Internal consistency of the CALLS was evaluated by means of item analysis and measured with Cronbach's coefficient alpha. Validity of the CALLS was assessed with respect to the MMSE (concurrent validity). Concurrent validity was measured with Pearson's r, after verifying the linear relationship between the CALLS and MMSE.

To assess construct validity, we conducted a principal component analysis of the CALLS battery, in which the covariance structure of the dependent variables was decomposed into orthogonal components by calculating the eigenvalues and eigenvectors of the data covariance matrix [42]. The eigenvalues were used in decision-making related to the number of orthogonal components used in subsequent analyses. Eigenvectors were used for determining the relationship between the original variables and subsequent components. Principal components were extracted using roots greater than one criterion and submitted to the Varimax procedure with an oblique rotation. The eigenvectors and eigenvalues transformed the initial variable space into a novel variable set of principal components.

Given the aim of concurrent validation, the same principal component analysis strategy was applied to the neuropsychological battery. Next correlations were calculated between the items in the CALLS battery and the component scores of the neuropsychological battery.

## Results

The means for each of the individual CALLS tests are displayed in Table 3. Of a possible 180 points, the CALLS total mean score for all participants was 104.4 (S.D. 19.9; range 50–150). Thirty-nine (18%) scored one standard deviation below the mean and 43 (20%) scored one standard deviation above the mean. As shown in Table 3, the CALLS scores are normally distributed. The distribution of CALLS total scores do not present ceiling/floor effects.

### Internal consistency

As shown in Table 4, the CALLS showed a high internal consistency, as measured by Cronbach's alpha (0.81). The Cronbach's alpha for the major factors were as follows: verbal learning and memory 0.88, processing speed 0.73, attention and working memory 0.56, verbal fluency and naming 0.46, and concept formation 0.18.

### Concurrent validity

The CALLS total score correlated moderately with the MMSE total score (Pearson's correlation, r = 0.60). Additional analyses of the relations between the MMSE total score and each of the CALLS domain factors revealed significant correlations: Verbal Learning and Memory (r = 0.41; p < 0.001); Processing Speed (r = 0.24; p < 0.001); Attention and Working Memory (r = 0.23; p < 0.001); Verbal Fluency and Naming (r = 0.38; p < 0.001); and Concept Formation (r = 0.33; p < 0.001).

### Construct validity

The principal component analysis resulted in five components with eigenvalues above one. These components accounted for 11 percent of the total matrix variance. The loadings are described in Table 4. The components were labeled as: a) verbal learning and memory (0.883); b) processing speed (0.731); c) attention and working memory (0.555); d) verbal fluency and naming (0.457); and e) concept formation (0.179).

Using the same principal component analysis strategy, the neuropsychological test battery yielded six components similar to the CALLS components. As shown in Table 5, the CALLS total score had largely moderate correlations (all statistically significant) with each of the neuropsychological tests. The strongest correlations were with Verbal Learning and Memory and Verbal Fluency and Naming. Weaker correlations tended to be with visuospatial (e.g. JLO) and non-verbal items (e.g. Facial Recognition). Further, the CALLS total score correlated with four neuropsychological testing components: Verbal Learning and Memory (r = 0.42; p < .0001), Verbal Fluency and Naming (r = 0.44; p < .0001), Episodic Memory for Contextual Information (r = 0.22; p < 0.0016), and Attention and Working Memory (r = 0.29; p < .0001). The CALLS did not correlate with visuospatial processing or non-verbal memory.

The CALLS battery also produced expected results for age and education. Older age was significantly correlated with lower CALLS scores (r = -0.35; p < 0.0001). Those with the highest education scored better on the total CALLS test than those with lower education.

Women scored higher than men on the CALLS total score (p = 0.0285), perhaps due to the higher proportion of verbal memory on the test. No CALLS score differences were found based on race or ethnicity.

Moderate intercorrelations were found between Simple Reaction Time and Verbal Fluency and Naming items such as Animal Naming (left ear 0.28, p < 0.0001; right ear 0.33, p < 0.001), F Words (left ear 0.21, p < 0.0027; right ear 0.19, p < 0.0048) and Word List Trial 1 (left ear 0.25, p < 0.003; right ear 0.27, p < 0.0001). The mean score for depression as measured by the CESD was 27.2 (S.D. 6.8) with a range of scores form 20 to 61. There were no significant correlations of the CALLS total or individual scores with depression.

## Discussion

The results of the current validation study suggest that the CALLS instrument is a valid measure for assessing cognitive function in an aging population. The linear correlation between the CALLS and the MMSE (Pearson r = 0.60; p < 0.05) revealed a moderate level of concurrent validity, despite different administration modalities (in-person administered versus telephone-administered). Additional analyses of the relations between the MMSE total score and each of the CALLS individual test items revealed significant correlations.

The CALLS total score was found to be strongly related to verbal learning and memory, verbal fluency and naming, attention and working memory, and episodic memory for contextual information. It was not associated with visuospatial or non-verbal factors from the neuropsychological battery. The majority of findings regarding the effect of age and education on the cognitive outcome were consistent with previous screens, and all results were in expected directions. These findings indeed suggest that the CALLS may be effectively used in place of standard in-person neuropsychological evaluations in situations where the CALLS would be more practical or where the standard in-person evaluations would be impractical to administer.

While further analytical work is required to assess the norms and predictive capacity of the CALLS, the potential clinical utility of the CALLS is reflected in its ability to perform as well as other tests or procedures. For example, the CALLS is well suited for assessing aspects measured by the MMSE, as well as some domains not well assessed by the MMSE. Additionally, the CALLS battery's 12-item word list with immediate and delayed conditions is significantly associated with the neuropsychological battery's verbal learning and memory component. Similarly, a strong association exists between the neuropsychological battery's verbal fluency and naming and the CALLS test component that includes semantic (animal naming) and phonemic (F words) fluency. Additionally, there was a noteworthy association between the CALLS version and the neuropsychological battery's version of digit span tests (forward and backward). The fact that these findings reveal such robust associations gives credence to the assertion that the CALLS battery validly measures these cognitive domains.

The CALLS battery provides unique measures of reaction time and processing speed. As a part of the cognitive progression, speed of processing is well documented to decline with age [43,44]. Moreover, the enhanced accuracy of timing assessment in the CALLS may make it more suitable for identifying deficits, especially when reduced processing speed and reaction time include delayed onset of responses and increased decision making times (i.e., reduced information processing speed). Further, in non-computerized assessments, there are some cases in which uncontrolled error margins between stimulus onset and actual stimulus display may result in the modeling of "noise" rather than veridical information [45]. Our results are preliminary, and while norms for different age groups will need to be established, the accuracy of our test is promising.

There were also moderate intercorrelations of reaction times with verbal learning and memory and verbal fluency and naming items in the CALLS battery and very small intercorrelations with the verbal fluency and naming items in the neuropsychological tests. These findings suggest the possible relationship of processing speed in retrieval of words from memory. They further suggest that failure to remember words in these tests may be more a function of slow speed in recalling words than of loss of verbal memory. Alternatively, this may be an indication that slow processing speed may impede sufficient verbal encoding for delayed word list recall.

Simple reaction time can also be a measure that distinguishes cognitively healthy from dementia groups [46]. The addition of response time choices found in the CALLS battery enhances the complexity of the response time measures and may increase sensitivity to screen for early dementia [46-48]. The addition of the adapted and shortened Center for Epidemiologic Studies Depression scale also provides a screen for depression, which is also known to slow processing speed.

Concept formation including word naming and similarities offers a simple test of concrete thinking and verbal expression. Each of these was correlated with the verbal memory components, and they uniquely address the ability to demonstrate abstract thinking and to identify concepts associated with commonly used words and the ease of retrieval of accurate words.

Although executive functioning did not comprise a unique component, elements of executive functioning are measured in the processing speed component with reaction times and in the individual tests of serial 7s and similarities. Each of these had strong factor loadings in the CALLS test. The failure to identify a specific, valid factor associated with executive functioning highlights the complexity of this construct and the difficulty of using a screening test to uniquely assess this domain [49].

The CALLS battery has a number of limitations. The CALLS battery requires the use of a telephone and there are no visuospatial or non-verbal tasks conducted. While there were modest yet significant correlations between the CALLS and the Trail Making Test (Parts A and B), as well as between the CALLS and Facial Recognition I and II, there was a lack of association with the full components. Given the fact that visuospatial deficits (problems with drawing, constructions, and orientation in their own surrounding) are among the earliest manifestations of Alzheimer's disease [50,51], the CALLS battery is faced with an important limitation. On the other hand, the lack of a visuospatial component in the CALLS battery may also be helpful in situations where a neuropsychological evaluation or screen needs to be administered to persons with severe visual deficits and specific physical disabilities.

While the sample had fairly equal representation for gender, age, and ethnicity, there were few with less than a high school education. Participants with lower education are generally more difficult to recruit and tend to have lower scores on cognitive tests. The small numbers in this group may have affected the distribution and results of the CALLS scores. The generalizability of results also can be affected by the relatively low response rate.

A further possible limitation of the CALLS battery is that it is not adapted for subjects where English is not their primary language. This resulted in 125, or 14 percent fewer possible subjects. While no CALLS score differences were found in the current study among ethnic and racial groups, it is possible that inclusion of these subjects would have altered that finding. Future studies should include a translated version of the CALLS for use with persons whose language is other than English.

An additional limitation is that the current study's data was insufficient to evaluate the validity of the CALLS battery for application to a sample inclusive of individuals with cognitive impairment ranging from mild (mild cognitive impairment) to severe (dementia). Although we did not specifically exclude anyone in our random sample based on cognitive status, we expect that the majority of our sample was cognitively unimpaired. Future studies should examine inclusion of patients affected by mild cognitive impairment, whether progressing or not to dementia. Hence, the CALLS battery should be applied to the study of prodromic cognitive deficits [52].

Despite these limitations, the CALLS battery has a number of strengths. Studies have shown that telephone testing of participants at home is not only reliable [53,54] but that screening at home rather than in the clinician's office may actually improve the performance of elderly subjects on these cognitive tests [55]. Further, the CALLS test provides a mechanism for the participant to select a hearing level comfortable to them that ensures appropriate volume for the test. One of the best features of the CALLS is its unique ability to measure simple and choice response times for each participant. Moreover, the thirty minutes required for the CALLS battery is more efficient and time preserving than most standard in-person neuropsychological evaluations. The two to four hour time period needed for face-to-face administration make such tests expensive and logistically unsuitable in most clinical and research settings. This is even more apparent with epidemiological studies. In addition to reduction of fatigue and increased accessibility, the CALLS battery reduces the need for expensive professional staff and locations. The utility of this instrument in large epidemiological studies is also likely increased by the fact that the test is administered via telephone with a computer interface, decreasing the need for training and validating test administrators at multiple sites.

## Conclusion

In summary, the CALLS battery was found to be a relatively brief, yet comprehensive standardized cognitive assessment tool with robust correlations to the more time-consuming and costly in-person neuropsychological battery. The test was scrupulously pre-tested and hierarchically staged to ensure that each step followed psychometrically valid procedures. These results show that multiple domains of cognitive functioning can be reliably assessed over the telephone. The CALLS instrument is a valid test with unique telephonic and computerized features that provides a unique potential to efficiently study cognitive function in large populations.

## Competing interests

The author(s) declare that they have no competing interests.

## Authors' contributions

VCC & JGB contributed to all aspects of design, analyses and implementation and interpretation of study, and drafts, revisions and critical review of paper.

TDP contributed to analyses and interpretation of study, and drafts, revisions and critical review of paper.

All have given final approval of this submission.

## Pre-publication history

The pre-publication history for this paper can be accessed here:



## Figures and Tables

**Table 1:** Selection of Sample

	Eligible Sample	Percent
	756	100
Bad phone numbers or maximum calls	48	6.35
Declined by postcard	158	20.9
Declined by telephone	333	44.0
Incomplete interviews	6	0.85
Completed interviews	211	27.9

Note: For all analyses N = 211.

**Table 2:** Demographic Characteristics of Sample

	**Participants**		**Non-Participants**		
			
**Demographic Category**	**Frequency** 211	**Percent ** 100%	**Frequency ** 697	**Percent ** 100%	**p**
***GENDER***					**n.s.**
Female	107	51%	348	50%	
Male	104	49%	349	50%	
***RACE/ETHNICITY***					**< .0001**
Asian/Pacific Islander	44	21%	194	28%	
African-Americans	48	23%	155	22%	
Hispanic	40	19%	251	28%	
White	76	36%	97	14%	
Other	3	1%	0	0	
***AGE***					**n.s.**
65–69	67	32%	262	38%	
70–74	59	28%	196	28%	
75–79	52	25%	132	19%	
80–84	22	10%	68	10%	
85+	11	5%	39	6%	
EDUCATION*					**NA**
< HS Graduate	14	7%	--	--	
HS Graduate	43	20%	--	--	
Some College	81	39%	--	--	
College+	72	34%	--	--	

Note: *With the exception of Education, the sample size for all analyses was N = 211. For Education, one case was missing and the resulting N = 210. NA = Education not available for non-participants.

**Table 3:** Descriptive Statistics of CALLS Interview

**Test**	**Mean Score**	**Standard Deviation**	**Range**
***Verbal Learning and Memory***			
Word List Trial 1	4.6	1.8	0–9
Word List Trial 2	6.4	2.1	1–12
Word List Trial 3	7.4	2.0	2–12
Word List Recognition	9.2	2.0	3–12
Word List Cued Recall	6.4	2.4	0–11
Word List Delayed Recall	5.7	2.8	0–11
***Processing Speed***			
Choice Reaction Time Decile	5.5	2.9	1–10
Simple Reaction Time Decile – Right ear	5.5	2.9	1–10
Simple Reaction Time Decile – Left ear	5.4	2.9	1–10
***Attention and Working Memory***			
DATE Score	3.6	0.9	0–5
Digit Span Backward – span	4.4	1.3	0–6
Digit Span Forward – span	6.2	0.9	4–7
***Verbal Fluency and Naming***			
President/VP Naming	3.0	1.0	0–4
F words -30 sec.	7.4	3.2	0–16
Animal Naming -30 sec.	11.5	3.4	0–23
Serial 7s	3.4	1.7	0–5
***Concept Formation***			
4 Word Naming	3.9	0.4	2–4
Similarities	4.8	2.1	0–7

Note: For all analyses N = 211.

**Table 4:** Factor Loadings of CALLS Test

**Rotated Component Matrix(a)**					
	**Verbal Learning & Memory**	**Processing Speed**	**Attention and Working Memory**	**Verbal Fluency and Naming**	**Concept Formation**

**Alpha**	**0.883**	**0.731**	**0.555**	**0.457**	**0.179**
					
Word List Trial 1	0.592				
Word List Trial 2	0.767				
Word Recognition	0.774				
Word List Trial 3	0.792				
Word List Cued Recall (Total)	0.829				
Word List Delayed Free Recall	0.834				
					
Choice RT Score Decile		0.559			
Simple RT Right Score Decile		0.865			
Simple RT Left Score Decile		0.893			
					
DATE Score			0.373		
Digit Span Backward (Span)			0.784		
Digit Span Forward (Span)			0.804		
					
President/VP Naming				0.799	
F Words (30 Seconds)				0.504	
Animal Naming (30 seconds)				0.456	
Serial 7's				0.496	
					
4 Word Naming					0.745
Similarities					0.732

Note: The extraction method was a Principal Component Analysis. The rotation method was Varimax with Kaiser Normalization. Rotation converged in 6 iterations. The CALLS Total coefficient alpha = 0.809. Choice RT = Choice Reaction Time; Simple RT = Simple Reaction Time.

**Table 5:** Correlations between CALLS Total Score and Neuropsychological Components

**Components-Items**	**r**	**p**
***Verbal Learning and Memory Component***	**0.42**	**< 0.0001**
CVLT Trials 1–5	0.57	< 0.0001
CVLT Trial 1 List A	0.36	< 0.0001
CVLT Trial 5	0.49	< 0.0001
CVLT List B	0.37	< 0.0001
CVLT Short Delay Free Recall	0.49	< 0.0001
CVLT Short Delay Cued Recall	0.44	< 0.0001
CVLT Long Delay Free Recall	0.47	< 0.0001
CVLT Long Delay Cued Recall	0.49	< 0.0001
CVLT Recognition	0.36	< 0.0001
		
***Verbal Fluency and Naming Component***	**0.44**	**< 0.0001**
Wechsler Test of Adult Reading	0.38	< 0.0001
Animal Naming	0.53	< 0.0001
F Word Total	0.51	< 0.0001
A Word Total	0.57	< 0.0001
S Word Total	0.49	< 0.0001
		
***Episodic Memory for Contextual Information Component ***	**0.22**	**0.0016**
Logical Memory I	0.41	< 0.0001
Logical Memory II	0.37	< 0.0001
Logical Memory Recognition	0.35	< 0.0001
Boston Naming Test	0.40	< 0.0001
		
***Attention & Working Memory Component***	**0.29**	**< 0.0001**
Digit Span Forward	0.27	< 0.0001
Digit Span Backward	0.38	< 0.0001
Letter-Number Sequencing	0.38	< 0.0001
Judgment of Line Orientation	0.18	0.0091
		
***Visuospatial Processing Component***	**-0.13**	**n.s.**
Trail Making Test Part A (time in seconds)	-0.33	< 0.0001
Trail Making Test Part B (time in seconds)	-.040	< 0.0001
		
***Non-Verbal Memory Component***	**0.09**	**n.s**
Faces I Immediate Recognition	0.19	0.0067
Faces II Delayed Recognition	0.26	0.0002

Note: For all analyses N = 211. CVLT = California Verbal Learning Test.
